# Factors influencing a common but neglected blood parasite prevalence in breeding populations of passerines

**DOI:** 10.1017/S0031182025000095

**Published:** 2025-02

**Authors:** Ashwin Kumar Saravana Bhavan Venkatachalam, Anna Kadlecová, Anna Kapustová, Magdalena Kulich Fialová, Jana Brzoňová, Miroslav Šálek, Milena Svobodová

**Affiliations:** 1 Department of Parasitology, Faculty of Science, Charles University, Praha, Czechia; 2 Department of Ecology, Faculty of Environmental Sciences, Czech University of Life Sciences, Praha, Czechia

**Keywords:** Acrocephalidae, Apicomplexa, avian haemoparasites, coccidia, host–parasite interaction, Lankesterellidae, Paridae, passerines

## Abstract

The occurrence of avian blood protists is affected by multiple factors that include the characteristics of the hosts, the vectors, the parasites, as well as the environmental factors. This study provides an insight into some of the factors that influence the prevalence of avian *Lankesterella*, neglected but common blood parasites in breeding populations of common passerines. The highest prevalences of *Lankesterella* infection were observed in 1 great tit (*Parus major*) population at 63%, 1 blue tit (*Cyanistes caeruleus*) population at 49% and a sedge warbler (*Acrocephalus schoenobaenus*) population at 33%. Prevalence was found to be significantly influenced by sampling site followed by host age, species and sex. Julian date had no significant effect on *Lankesterella* prevalence. Prevalence data from different sampling sites can reveal different patterns and should be combined critically. Higher prevalence in adults suggest that the infections are chronic, which helps the parasite to persist in host populations. The differences between sexes might be related to different exposure to the transmitting vectors (e. g., mites or mosquitoes) during breeding.

## Graphical abstract

**Figure:**
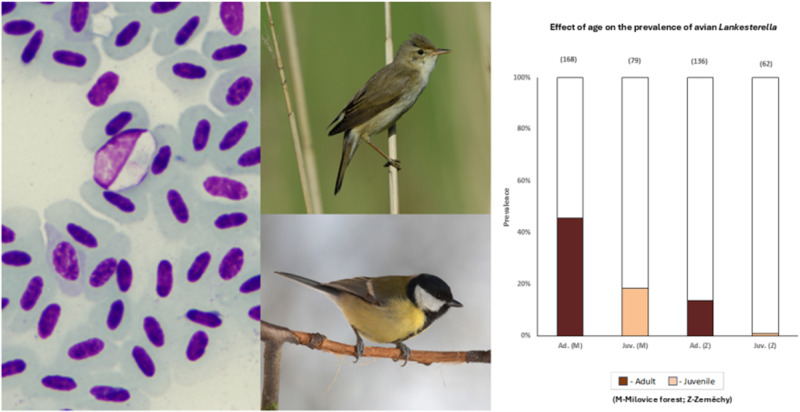

## Introduction

Avian blood protists are frequently found in most species of passerines. Those belonging to Apicomplexa are represented either by intracellular, notorious haemosporidian parasites such as *Plasmodium, Haemoproteus* and *Leucocytozoon*, or by neglected coccidian parasites such as *Hepatozoon, Isospora* and *Lankesterella*. Based on research done in reptiles and amphibians, the genus *Lankesterella* is considered heteroxenous (Desser, 1993; Megía-Palma et al., 2016). Infective sporozoites circulating in blood cells are taken up by bloodsucking invertebrate vectors (leeches, mites or mosquitoes), but no replication has been observed in the invertebrate hosts (Desser, 1993). *Lankesterella* are reported in blood of various avian species and confirmed by barcoding in several passerine genera (Merino et al., 2006; Biedrzycka et al., 2013; Martínez et al., 2018; Chagas et al., 2021a, b; Venkatachalam et al., 2023). It is evident that *Lankesterella* is common at least in some populations of passerines, but despite this, we have limited information about its occurrence and prevalence. In a phylogenetic context, *Lankesterella* does not belong to the well-known haemosporidian parasites (Adl et al., 2018). Consequently, avian *Lankesterella* is somewhat mysterious, and any data concerning this parasite genus are exceptionally valuable.

Host species is an important factor that can influence blood parasite prevalence in birds. *Lankesterella* lineages from sedge warbler (*Acrocephalus schoenobaenus*) were found to be highly host specific when compared to the other parasite lineages of warblers; tit genera have been shown to have their own specific lineages (Venkatachalam et al., 2023). At present, data on *Lankesterella* prevalences are scarce, let alone the knowledge about the factors that could influence their prevalence. Only a few studies have been done so far, focusing either on a single host species or investigating non-breeding populations. Prevalences can be high: 31% of adult blue tits (*Cyanistes caeruleus*), 47% of adult sedge warblers and 20% of snow bunting (*Plectrophenax nivalis*) nestlings were found to be infected (Merino et al., 2006; Biedrzycka et al., 2013; Martínez et al., 2018). Prevalence of *Lankesterella* in adult (after hatch year) and juvenile (hatch year) migrating warblers (*Acrocephalus* spp.) was 16% and 7%, respectively, suggesting and effect of age (Chagas et al., 2021a). In some species, however, prevalence can be as low as 2% (adult common house martin (*Delichon urbicum*)), or *Lankesterella* are not detected at all (Chagas et al., 2021a). Hence, studies comparing prevalences in multiple host species and in breeding populations would be valuable to assess the influencing factors. Various factors affect the prevalence of blood parasites in their hosts. Since no studies are available for avian *Lankesterella* yet, we must rely on other blood protists for assessing the factors potentially influencing its prevalences.

Sampling site may be an important factor, as it may vary in the abundance and species of vectors, or the number of potential hosts. Indeed, significant variations in haemosporidian *(Plasmodium, Haemoproteus and Leucocytozoon)* prevalence based on sampling site were observed in different avian species (Emmenegger et al., 2020; Grieves et al., 2023). Haemosporidian infection prevalences can also emerge from regional-scale habitat variation (Fecchio et al., 2021). Parasite infection prevalences often vary with the age of infected individuals (Slowinski et al., 2022). Usually, adults have higher haemosporidian prevalences compared to younger birds, probably due to longer exposure in combination with persistent infections (Valkiūnas, 2005; Fecchio et al., 2015; Svobodová et al., 2015; Wilkinson et al., 2016; Huang et al., 2020; Yang et al., 2023). Sex-biased parasitism is usually attributed to differences in hormone levels. The male sex hormone, testosterone, can suppress humoral immunity in males whereas both testosterone and oestrogen can reduce cell-mediated immunity and at the same time boost humoral immunity (Zuk and McKean, 1996; McCurdy et al., 1998). Males were found to be more likely infected with haemosporidian parasites compared to females (Calero-Riestra and García, 2016; Rodriguez et al., 2021; Che-Ajuyo et al., 2023; Grieves et al., 2023). However, sex-biased parasitism need not be attributed solely to testosterone since its manipulation did not increase infection probability (McCurdy et al., 1998; Slowinski et al., 2022). Females can have higher prevalences in case of opposite sexual dimorphism (Svobodová et al., 2023). Besides physiological differences between sexes, exposure to parasites may play an important role; e.g. incubating females of species with open nests are more prone to parasites transmitted by flying bloodsucking vectors since not all vectors enter cavities (Votýpka et al., 2009). Moreover, natural cavities and nest boxes differ in their microclimate and suitability for potential vectors like avian fleas and mites (Maziarz et al., 2017).

Julian date can be an important factor that influences blood parasite prevalence in avian species. Specifically, the breeding season can be a period of increased physical demand in birds, causing stress resulting in immunosuppression and thus a higher susceptibility to, or relapses of, previous infections (Norris and Evans, 2000; Valkiūnas et al., 2004; Granthon and Williams, 2017).

The aim of this study was to determine the factors that influence the prevalence of avian *Lankesterella* in passerines. For our study, we selected 3 species of cavity nesting, resident/short distant migrant species of tits, i.e. great tit (*Parus major*), blue tit and marsh tit (*Poecile palustris*), family Paridae, and 3 species of open nesting, long distance migrating passerines from the family Acrocephalidae, i.e. sedge warbler, reed warbler (*Acrocephalus scirpaceus*) and marsh warbler (*A. palustris*) (Storchová and Hořák, 2018). All these species feed on insects and other arthropods while tits are also granivorous, and marsh tits feed additionally on fruits (Storchová and Hořák, 2018). Tits are primarily woodland species and *Acrocephalus* warblers mostly inhabit reedbeds or swampland. In the studied model species, both male and female reed and marsh warblers participate in egg incubation, unlike in other species where only females incubate (Storchová and Hořák, 2018).

The model species are known for *Lankesterella* occurrence and were selected based on their abundance and sympatric occurrence in the studied area. Moreover, their blood parasites are readily used as models for studying host–parasite interactions. We hypothesized that (i) adults are more likely to be infected due to prolonged exposure to the parasite which persists in its host after infection, (ii) males are more likely to be infected (e.g. due to higher testosterone levels); alternatively, incubating females might be more prone to infection (due to increased exposure to vectors like mites and mosquitoes at nests) and, (iii) prevalences differ between host taxa (families) at the same sites due to different life history traits of the hosts.

## Methodology

### Field work and blood sampling

Birds were trapped and ringed during the breeding season (April–July) from 2014 to 2022 using mist nets or in nest boxes as described in Fialová et al. (2021) and Venkatachalam et al. (2023), at 2 localities in Czechia, namely, Zeměchy (50.230374 N, 14.278040 E, with reed/shrub habitat with a little stream) and Milovice forest (48.825200 N, 16.686286 E, game reserve consisting of dry oak forest with multiple clear-cuts). All bird captures and manipulations were carried out by licensed workers. The species, sex and age were determined for each individual. Blood was taken from the metatarsus vein articulation (*vena metatarsalis plantaris superficialis media*); 10–20 μl of blood was stored in 96% ethanol for further use. Blood sampling was carried out under permits 50982/ENV/14-2961/630/14 and MZP/2019/630/1081 of the Ministry of the Environment. Tit and warbler species were both caught in Zeměchy using mist nets whereas only tits were caught in Milovice forest, females from nest boxes and both sexes by setting up mist nests at the nest boxes or at watering sites.

### Parasite detection methods and host sexing

When available, about 25 yearlings, 25 adult males and 25 adult females from each host species were used for the analysis. In case of more blood samples available in the respective categories, we randomly selected samples from different sampling years and months to avoid bias. DNA from bird blood was isolated, a nested Polymerase Chain Reaction (PCR) protocol targeting the coccidian Small SubUnit (SSU) rRNA gene was used for *Lankesterella* detection, and positive samples were sequenced using Sanger sequencing and barcoded using the Basic Local Alignment and Search Tool (BLAST) algorithm in the National Center for Biotechnology Information (NCBI) database (Venkatachalam et al., 2023). To avoid cross-contamination, DNA from a single host species was used in individual PCR runs that contained no more than 16 samples. A negative control (PCR H_2_O) was used for each PCR run. DNA from blood positive for *Lankesterella* was used as a positive control. A molecular sexing protocol (Griffiths et al., 1998) was used in cases where sex could not be assessed (approximately 15% of adult warblers before/after breeding).

### Statistical analysis

Statistical analysis was performed using R studio software (version 4.1.2, R Development Core Team, 2021) using the lmerTest package (Kuznetsova et al., 2017). Generalized linear models with binomial response (infection - yes/no) were used to assess the fixed effects of age (adults and yearlings), site (Zeměchy and Milovice forest), sex (males and females), bird species and sampling date entered as centred Julian date (84–205) on *Lankesterella* infection status. We implemented 2 separate models to test the effect of age and the other for sex, because data on both variables were not simultaneously available to analyse them in a single, comprehensive model. To increase the robustness of the tested dataset, the species were divided into 2 families with different life histories (Paridae and Acrocephalidae). Caught birds were aged and categorized as adults (hatched in the previous year or older) or yearlings (hatched in the current year). A dataset containing a total of 1032 samples including retraps (316 repeatedly sampled individuals) was used for the analysis. To avoid pseudoreplication (retraps), the function ‘Duplicated()’ was used to exclude the repeatedly sampled individuals at a random level.

## Results

The presence of *Lankesterella* was tested in 1032 individuals caught between 2014 and 2022. This includes 459 adults (284 males and 175 females) and 128 yearlings of *Acrocephalus* spp., and 304 adults (127 males and 177 females) and 141 yearlings of *Parus* s. l. spp. (*Parus, Cyanistes, and Poecile*). The prevalences of *Lankesterella* in individual host species, as well as prevalences according to age, sex and site, are given in Table 1. Overall, 6% (62/1032) of the samples were barcoded as *Isospora*; these samples were treated as *Lankesterella*-negative. Unresolved sequences were excluded from the analysis.

**Table 1.** *Lankesterella* prevalences in model passerine species, categorized by host species, site, age and sex. Numbers in parentheses indicate infected individuals and the total number of individuals tested

Host	Site	Adults	Yearlings	Males	Females
*C. caeruleus*	Milovice forest	0·49 (31/63)	0·26 (8/30)	0·63 (17/27)	0·38 (14/36)
*P. major*		0·63 (51/81)	0·16 (4/25)	0·64 (18/28)	0·62 (33/53)
*P. palustris*		0·25 (6/24)	0·12 (3/24)	0·22 (2/9)	0·26 (4/15)
*C. caeruleus*	Zeměchy	0·23 (15/64)	0 (0/30)	0·16 (4/25)	0·28 (11/39)
*P. major*		0·17 (11/64)	0·03 (1/32)	0·13 (5/36)	0·21 (6/28)
*P. palustris*		0 (0/8)	N/A	0 (0/2)	0 (0/6)
*A. palustris*	Zeměchy	0·06 (13/186)	0·01(1/67)	0·04 (5/106)	0·10 (8/80)
*A. schoenobaenus*		0·33 (24/72)	0·09 (3/32)	0·25 (11/43)	0·44 (13/29)
*A. scirpaceus*		0·16 (32/201)	0·06 (2/29)	0·10 (14/135)	0·27 (18/66)

**Figure 1.**
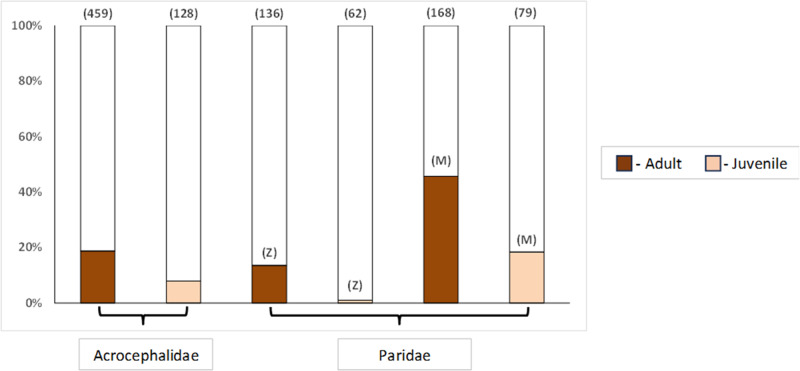
*Lankesterella* prevalences of adults and juvenile individuals in the Acrocephalidae family (*A. schoenobaenus, A. palustris* and *A. scirpaceus*) and the Paridae family (*C. caeruleus, P. major* and *P. palustris*) from Zeměchy (**Z**) and Milovice forest (**M**), respectively. Number of individuals is shown above the columns.

**Figure 2.**
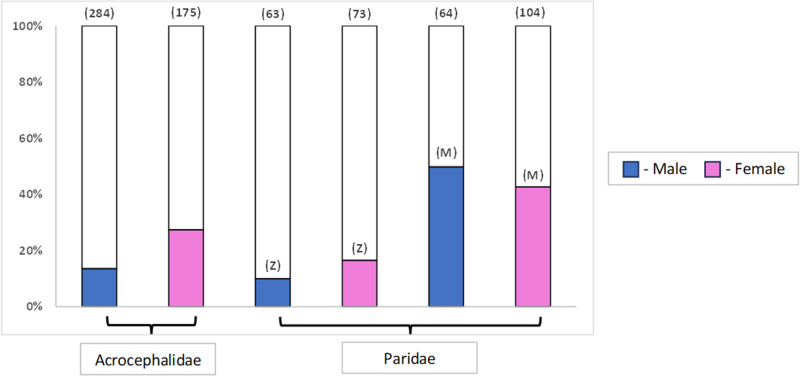
*Lankesterella* prevalences of male and female individuals in the Acrocephalidae family (*A. schoenobaenus, A. palustris* and *A. scirpaceus*) and the Paridae family (*C. caeruleus, P. major* and *P. palustris*) from Zeměchy (**Z**) and Milovice forest (**M**), respectively. Number of individuals is shown above the columns.

Overall, prevalence in adults was consistently higher than in yearlings in both the respective species or family and site combinations (Table 1; Figure 1). Specifically, the highest prevalence of *Lankesterella* in adults was found in great tits (63%) followed by blue tits (49%), both in Milovice forest. Among warblers, the prevalence of *Lankesterella* in adults was the highest in sedge warblers (33%, see supplementary figures (i, ii) for detailed graphs on the species level). As for sex, the prevalences between males and females differed as well. In Zeměchy, female birds from both tested families had a higher prevalence of *Lankesterella* infections than males, whereas in Milovice forest, the trend was opposite in Paridae (Figure 2), primarily due to the Blue Tit *Cyanistes caeruleus* (Table 1; see supplementary figures (iii, iv) for detailed graphs on the species level).

### *The effect of host site, age, family, Julian date and the interaction of host age and family on* Lankesterella *infections*

We tested the effect of age (adults vs yearlings) in all species and sites (Table 2). Species were merged as families (Acrocephalidae and Paridae) to make the dataset more robust. The model showed that birds from Milovice forest had a higher prevalence compared to Zeměchy (*p* < 0·001) and adults had a higher prevalence compared to yearlings (Table 1; Figure 1). No significant effect of Julian date was observed. As for the interaction of age and family, adults of the family Paridae are more likely to be infected (Table 2, Figure 1).

**Table 2.** The effect of age (adults vs yearlings), site (Zeměchy vs Milovice forest), family, Julian date, and the interaction of age and family on *Lankesterella* infections in passerine hosts (* indicates statistical significance)

Factor	Estimate	**SE**	*z*-value	*p*-value
Intercept	− 0·72	1·02	− 0·71	0·476
Adults	0·57	0·42	1·35	0·176
Family-Paridae	− 0·69	0·50	− 1·38	0·166
Zeměchy	− 1·69	0·27	− 6·23	< 0·001*
Julian date	− 0·00	0·00	− 0·14	0·885
Adults: Family-Paridae	1·11	0·49	2·26	0·023*

### *The effect of host site, sex, family, Julian date and the interaction of host sex and family on* Lankesterella *infections*

We tested the effect of host sex (males vs females) and the interaction of sex with family on a subset of adult birds across all genera and sites (Table 3). Birds from Milovice forest have higher prevalences (*p* < 0·001). Beyond the effects detected by the previous model, the effect of sex in females as an individual level factor was significant in most species (*p* = 0·007). As for the interaction of host sex and family, we see that males of the family Paridae are more likely to be infected (Table 3, Figure 2).

**Table 3.** The effect of sex (males vs females), site (Zeměchy vs Milovice forest), family, Julian date, and the interaction of sex and family on *Lankesterella* infections in Parus s. l. spp. (* indicates statistical significance)

Factor	Estimate	**SE**	*z*-value	*p*-value
Intercept	0·69	0·89	0·77	0·441
Males	− 0·99	0·36	− 2·69	0·007*
Family-Paridae	− 0·04	0·36	− 0·12	0·904
Zeměchy	− 1·56	0·28	− 5·43	< 0·001*
Julian date	− 0·00	0·00	− 0·70	0·484
Males: Family-Paridae	0·98	0·45	2·17	0·029*

## Discussion

In our study, *Lankesterella* parasites were readily found in the blood of the studied host populations, but with high variation between tested categories in prevalences. The occurrence of *Lankesterella* in host blood can considerably differ depending on the host species. At the host family level, Paridae were more infected with *Lankesterella* than Acrocephalidae, but there were also differences between the species within families. Adult individuals of great tit and blue tit populations in 1 of 2 studied localities had very high prevalences of *Lankesterella* (63% and 49%, respectively; see Table 1) compared to the third related species (*P. palustris*), which had surprisingly the lowest prevalence; no other data are available for great tit but the prevalence in Spanish blue tits was 31% (Merino et al., 2006). Sedge warbler had the highest prevalence among the adults of *Acrocephalus* spp. (33%) (Table 1). High prevalence was detected in sedge warbler in other studies as well, reaching 47% in adult birds in Poland, and 33% in Lithuania (Biedrzycka et al., 2013; Chagas et al., 2021a). Blood parasite prevalence therefore considerably varies depending on the host species, even in birds that occur at the same sites and have similar exposure to potential vectors.

Host–parasite relationships are influenced by multiple factors (Ellis et al., 2020); since there is a considerable degree of *Lankesterella* host specificity at the genus level (Venkatachalam et al., 2023), it is hard to separate the influence of host and parasite life history traits. The most significant factor influencing prevalence in this study was the sampling site, followed by host age and sex to some degree. However, the influences of these factors should be interpreted with caution, as age and sex had to be analysed in separate models. This was since, although yearlings can be sexed by genotype, sex differences are not yet phenotypically pronounced. An interesting pattern was revealed among the studied warbler species; sedge warblers host lineages which are species-specific while other warbler species share a different set of lineages (Chagas et al., 2021a; Venkatachalam et al., 2023). Sedge warbler lineages have the highest prevalence (33%) among warbler species when assessed at the host species level, while prevalence across all *Acrocephalus* spp. is higher for the generalist lineages (10% vs 5% overall prevalences) (Chagas et al., 2021a). The specialist parasite thus reaches higher prevalence in its specific host while the generalist can profit from higher host availability (see Drovetski et al., 2014).

There was a significant difference in prevalences of *Lankesterella* parasites based on the sampling sites (Tables 1–3); Milovice forest had an overall higher prevalence of *Lankesterella* in the respective host species. Previous studies on avian haemosporidian parasites showed that location/sampling site is an important factor influencing prevalences (Fecchio et al., 2021; Grieves et al., 2023; Yusupova et al., 2023). A recent comprehensive study focusing on *Lankesterella* revealed the highest prevalence in sedge warbler among our model species, while prevalences in tits were negligible; however, samples were collected across multiple European localities, parasites were detected in multiple tissues, and the age of the birds was not specified (Keckeisen et al., 2024), making the comparison with our data difficult. The effects of habitat and breeding habits are not mutually exclusive; since the vectors of avian *Lankesterella* are not known, we can only speculate about the potential impact on transmission.

Age has a significant effect on *Lankesterella* infection status, with adult individuals consistently more infected than yearlings (Table 2; Figure 1). The positive correlation of host age and parasite prevalence was found in several host–parasite associations (Norris et al., 1994; Svobodová et al., 2015). Older individuals tend to have a higher risk of parasite infection due to cumulative exposure or potential immunosenescence (Wood et al., 2007; Knowles et al., 2011; Synek et al., 2016; Eastwood et al., 2019). Although yearlings had lower prevalences in our study, the presence of *Lankesterella* confirms ongoing on-site transmission. The higher prevalences in adults as a consequence of parasite persistence might explain an apparent discrepancy in prevalences: adult blue tits in 2 Spanish studies had prevalences of 31% and 9%, respectively; however, only after-hatch year birds were sampled in the latter study (Merino et al., 2006; Castaño‐Vázquez and Merino, 2022).

Sex influenced the prevalence of *Lankesterella* parasites. The model indicated that females were significantly more likely to be infected with *Lankesterella* in the majority of cases (Table 3; Figure 2). Sex is usually an important intrinsic factor associated with increased susceptibility to parasite infections (McCurdy et al., 1998). In various genera of lizards, the occurrence of *Lankesterella* was higher in females as well (Drechsler et al., 2021) (but see the exception of the Western fence lizard (*Sceloporus occidentalis*) where males were more infected) (Megía-Palma et al., 2018). In the case of haemosporidian infections in birds, several studies have found significant influence of host sex based on the parasite species found in the host (Rodriguez et al., 2021; Grieves et al., 2023; Yusupova et al., 2023). The prevalence of 3 blood parasite genera was higher in female Eurasian sparrowhawk (*Accipiter nisus*) supposedly due to higher exposure at nest, either during breeding or already at the nestling stage (Svobodová et al., 2023). Differences like nesting behaviour among the different hosts can lead to different levels of *Lankesterella* prevalences. Differential exposure of vectors can arise from unequal time spent at the nest during egg incubation and nestling care (Zuk and McKean, 1996).

No effect of Julian date on *Lankesterella* prevalences was observed in our study. Although adults may exhibit chronic infections, juvenile prevalence is expected to increase in the course of the season. To exclude the effect of chronicity on parasite infections, we tested the effect of Julian date with juveniles only, and no significant effect was observed (data not shown). The absence of the Julian date effect can thus be caused by a short sampling period confined to the breeding season. Alternatively, transmission can occur mainly at the nestling stage. The prevalence of haemosporidia in nestlings of 2 species of raptors significantly increased with Julian date (Svobodová et al., 2015). There are not many studies that have Julian date as a factor influencing haemosporidian prevalences. However, many studies showed that prevalence of avian haemosporidians increases over the breeding season (Ventim et al., 2012; Grieves et al., 2023). This can be due to vector availability and reduced host immunocompetence due to reproduction stress and energy investment (Schultz et al., 2010; Ventim et al., 2012). A longer sampling period extended to non-breeding season might reveal the effect of Julian date on *Lankesterella* prevalences in passerine hosts; however, since Czech populations of warblers begin migration already in the second half of July, and yearling tits disperse (Cepák et al., 2008), this applies rather to the strictly resident species than to our model hosts.

## Conclusion

We found substantial variation in *Lankesterella* parasite prevalence between the 2 families and among 6 model species of these passerine families. From the statistical models, we found that the most important factor influencing *Lankesterella* prevalence in the hosts was the sampling site, followed by host age and sex. Adult individuals have higher prevalences, probably due to parasite persistence. Moreover, females tend to have a higher prevalence of infection, which may be due to greater exposure to vectors during incubation. No effect of Julian date was revealed. The presence of *Lankesterella* in yearlings confirms on-site transmission. This study highlights the importance of the various ecological factors shaping avian *Lankesterella* parasite prevalences; in particular, the most important effect of sampling site warns against uncritical merging of data derived from multiple host populations when assessing prevalence.

## Supporting information

Saravana Bhavan Venkatachalam et al. supplementary material Saravana Bhavan Venkatachalam et al. supplementary material

## Data Availability

Data used for statistical analysis available on request.

## Supplementary material

The supplementary material for this article can be found at https://doi.org/10.1017/S0031182025000095.

## References

[ref1] Adl SM, Bass D, Lane CE, Lukeš J, Schoch CL, Smirnov A, Agatha S, Berney C, Brown MW, Burki F, Cárdenas P, Čepička I, Chistyakova L, del CJ, Dunthorn M, Edvardsen B, Eglit Y, Guillou L, Hampl V, Heiss AA, Hoppenrath M, James TY, Karnkowska A, Karpov S, Kim E, Kolisko M, Kudryavtsev A, Lahr DJ, Lara E and Le Gall L (2018) Revisions to the Classification, nomenclature, and diversity of Eukaryotes. *Journal of Eukaryotic Microbiology* 66, 4–119. doi:10.1111/jeu.12691. PMC6492006 30257078

[ref2] Biedrzycka A, Kloch A, Migalska M and Bielański W (2013) Molecular characterization of putative *Hepatozoon* sp. from the sedge warbler (*Acrocephalus schoenobaenus*). *Parasitology* 140, 695–698. doi:10.1017/S0031182012002004. 23363535

[ref3] Calero-Riestra M and García JT (2016) Sex-dependent differences in avian malaria prevalence and consequences of infections on nestling growth and adult condition in the Tawny pipit, *Anthus campestris*. *Malaria Journal* 15, 1–11. 27001667 10.1186/s12936-016-1220-y PMC4802721

[ref4] Castaño‐Vázquez F and Merino S (2022) Differential effects of environmental climatic variables on parasite abundances in blue tit nests during a decade. *Integrative Zoology* 17, 511–529. 34971472 10.1111/1749-4877.12625 PMC9543696

[ref5] Cepák J, Klvaña P, Škopek J, Schropfer L, Jelínek M, Hořák D, Formánek J and Zárybnický J eds (2008) *Atlas Migrace Ptáků České Republiky a Slovenska*. Praha: Aventinum.

[ref6] Chagas CRF, Binkienė R and Valkiūnas G (2021b) Description and molecular characterization of two species of avian blood parasites, with remarks on circadian rhythms of avian haematozoa infections. *Animals* 11, 3490. doi:10.3390/ani11123490. 34944267 PMC8698112

[ref7] Chagas CRF, Harl J, Preikša V, Bukauskaitė D, Ilgūnas M, Weissenböck H and Valkiūnas G (2021a) *Lankesterella* (Apicomplexa, Lankesterellidae) blood parasites of Passeriform birds: Prevalence, molecular and morphological characterization, with notes on sporozoite persistence in vivo and development in vitro. *Animals* 11, 1451. doi:10.3390/ANI11051451. 34070187 PMC8158525

[ref8] Che-Ajuyo NMA, Liu B, Deng Z, Rao X, Dong L and Liang W (2023) Sex-biased, but not plumage color-based, prevalence of haemosporidian parasites in free-range chickens. *Parasitology International* 93, 102722. 36529451 10.1016/j.parint.2022.102722

[ref9] Desser SS (1993) The Haemogregarinidae and Lankesterellidae. New York, USA: Parasitic protozoa, Academic Press, 4(2), 265–269. doi:10.1016/B9780-12-426014-6.50009-0

[ref10] Drechsler RM, Belliure J and Megía-Palma R (2021) Phenological and intrinsic predictors of mite and haemacoccidian infection dynamics in a Mediterranean community of lizards. *Parasitology* 148, 1328–1338. 34078494 10.1017/S0031182021000858 PMC8383277

[ref11] Drovetski SV, Aghayan SA, Mata VA, Lopes RJ, Mode NA, Harvey JA and Voelker G (2014) Does the niche breadth or trade‐off hypothesis explain the abundance–occupancy relationship in avian Haemosporidia? *Molecular Ecology* 23, 3322–3329. 24689968 10.1111/mec.12744

[ref12] Eastwood JR, Peacock L, Hall ML, Roast M, Murphy SA, da Silva AG and Peters A (2019) Persistent low avian malaria in a tropical species despite high community prevalence. *International Journal for Parasitology: Parasites and Wildlife* 8, 88–93. 30723669 10.1016/j.ijppaw.2019.01.001 PMC6350384

[ref13] Ellis VA, Huang X, Westerdahl H, Jönsson J, Hasselquist D, Neto JM, Nilsson J-Å, Nilsson J, Hegemann A, Hellgren O and Bensch S (2020) Explaining prevalence, diversity and host specificity in a community of avian haemosporidian parasites. *Oikos* 129, 1314–1329.

[ref14] Emmenegger T, Alves JA, Rocha AD, Costa JS, Schmid R, Schulze M and Hahn S (2020) Population- and age-specific patterns of haemosporidian assemblages and infection levels in European bee-eaters (*Merops apiaster*). *International Journal for Parasitology* 50, 1125–1131. 32866492 10.1016/j.ijpara.2020.07.005

[ref15] Fecchio A, Clark NJ, Bell JA, Skeen HR, Lutz HL, De La Torre GM, Vaughan JA, Tkach VV, Schunck F, Ferreira FC, Braga ÉM, Lugarini C, Wamiti W, Dispoto JH, Galen SC, Kirchgatter K, Sagario MC, Cueto VR, González-Acuña D and Wells K (2021) Global drivers of avian haemosporidian infections vary across zoogeographical regions. *Global Ecology and Biogeography* 30, 2393–2406. doi:10.1111/geb.13390.

[ref16] Fecchio A, Lima MR, Silveira P, Ribas ACA, Caparroz R and Marini MÂ (2015) Age, but not sex and seasonality, influence Haemosporida prevalence in White-banded Tanagers (*Neothraupis fasciata*) from central Brazil. *Canadian Journal of Zoology* 93, 71–77. doi:10.1139/cjz-2014-0119.

[ref17] Fialová M, Santolíková A, Brotánková A, Brzoňová J and Svobodová M (2021) Complete life cycle of *Trypanosoma thomasbancrofti*, an avian trypanosome transmitted by Culicine mosquitoes. *Microorganisms* 9, 2101. doi:10.3390/microorganisms9102101. 34683422 PMC8539158

[ref18] Granthon C and Williams DA (2017) Avian malaria, body condition, and blood parameters in four species of songbirds. *The Wilson Journal of Ornithology* 129, 492–508.

[ref19] Grieves LA, Balogh L, Kelly TR and MacDougall-Shackleton EA (2023) Haemosporidian infection prevalence varies temporally and spatially and *Leucocytozoon* infections are male biased in Song Sparrows. *Ornithology* 140, ukad008. doi:10.1093/ornithology/ukad008.

[ref20] Griffiths R, Double MC, Orr K and Dawson RJ (1998) A DNA test to sex most birds. *Molecular Ecology* 7, 1071–1075. doi:10.1046/j.1365-294x.1998.00389.x. 9711866

[ref21] Huang X, Jönsson J and Bensch S (2020) Persistence of avian haemosporidians in the wild: A case study to illustrate seasonal infection patterns in relation to host life stages. *International Journal for Parasitology* 50, 611–619. 32598873 10.1016/j.ijpara.2020.05.006

[ref22] Keckeisen C, Šujanová A, Himmel T, Matt J, Nedorost N, Chagas CRF, Weissenböck H and Harl J (2024) *Isospora* and *Lankesterella* parasites (Eimeriidae, Apicomplexa) of Passeriform birds in Europe: Infection rates, phylogeny, and pathogenicity. *Pathogens* 13, 337. doi:10.3390/pathogens13040337. 38668292 PMC11053544

[ref23] Knowles SCL, Wood MJ, Alves R, Wilkin TA, Bensch S and Sheldon BC (2011) Molecular epidemiology of malaria prevalence and parasitaemia in a wild bird population. *Molecular Ecology* 20, 1062. 21073677 10.1111/j.1365-294X.2010.04909.x

[ref24] Kuznetsova A, Brockhoff PB and Christensen RHB (2017) lmerTest Package: Tests in linear mixed effects models. *Journal of Statistical Software* 82, 1–26. doi:10.18637/jss.v082.i13.

[ref25] Martínez J, Merino S, Badás EP, Almazán L, Moksnes A and Barbosa A (2018) Hemoparasites and immunological parameters in snow bunting (*Plectrophenax nivalis*) nestlings. *Polar Biology* 41, 1855–1866. doi:10.1007/s00300-018-2327-0.

[ref26] Maziarz M, Broughton RK and Wesołowski T (2017) Microclimate in tree cavities and nest-boxes: Implications for hole-nesting birds. *Forest Ecology and Management* 389, 306–313.

[ref27] McCurdy DG, Shutler D, Mullie A and Forbes MR (1998) Sex-biased parasitism of avian hosts: Relations to blood parasite taxon and mating system. *Oikos* 82, 303–312. doi:10.2307/3546970.

[ref28] Megía-Palma R, Martínez J, Nasri I, Cuervo JJ, Martín J, Acevedo I, Belliure J, Ortega J, García-Roa R, Selmi S and Merino S (2016) Phylogenetic relationships of *Isospora, Lankesterella*, and *Caryospora* species (Apicomplexa: Eimeriidae) infecting lizards. *Organisms Diversity and Evolution* 16, 275–288. doi:10.1007/s13127-015-0253-3.

[ref29] Megía-Palma R, Paranjpe D, Reguera S, Martínez J, Cooper RD, Blaimont P, Merino S and Sinervo B (2018) Multiple color patches and parasites in *Sceloporus occidentalis*: Differential relationships by sex and infection. *Current Zoology* 64, 703–711. doi:10.1093/cz/zoy007. 30538729 PMC6280098

[ref30] Merino S, Martínez J, Martínez-de la Puente J, Criado-Fornelio Á, Tomás G, Morales J and García-Fraile S (2006) Molecular characterization of the 18S rDNA gene of an avian *Hepatozoon* reveals that it is closely related to *Lankesterella*. *Journal of Parasitology* 92, 1330–1335. 17304816 10.1645/GE-860R.1

[ref31] Norris K, Anwar M and Read AF (1994) Reproductive effort influences the prevalence of haematozoan parasites in great tits. *Journal of Animal Ecology* 63, 601–610. doi:10.2307/5226.

[ref32] Norris K and Evans MR (2000) Ecological immunology: Life history trade-offs and immune defence in birds. *Behavioral Ecology* 11, 19–26. doi:10.1093/beheco/11.1.19.

[ref33] R Core Team (2021) *R: A language and environment for statistical computing*. R Foundation for Statistical Computing. https://www.R-project.org/.

[ref34] Rodriguez MD, Doherty PF, Piaggio AJ and Huyvaert KP (2021) Sex and nest type influence avian blood parasite prevalence in a high-elevation bird community. *Parasites and Vectors* 14, 145. doi:10.1186/s13071-021-04612-w. 33685479 PMC7938522

[ref35] Schultz A, Underhill LG, Earlé RA and Underhill G (2010) Infection prevalence and absence of positive correlation between avian haemosporidian parasites, mass and body condition in the Cape Weaver *Ploceus capensis*. *Ostrich* 81, 69–76. doi:10.2989/00306521003690630.

[ref36] Slowinski SP, Geissler AJ, Gerlach N, Heidinger BJ and Ketterson ED (2022) The probability of being infected with haemosporidian parasites increases with host age but is not affected by experimental testosterone elevation in a wild songbird. *Journal of Avian Biology*. doi:10.1111/jav.02819.

[ref37] Storchová L and Hořák D (2018) Life-history characteristics of European birds. *Global Ecology and Biogeography* 27, 400–406. doi:10.1111/geb.12709.

[ref38] Svobodová M, Čepička I, Zídková L, Kassahun A, Votýpka J, Peške L, Hrazdilová K, Brzoňová J, Voříšek P and Weidinger K (2023) Blood parasites (*Trypanosoma, Leucocytozoon, Haemoproteus*) in the Eurasian sparrowhawk (*Accipiter nisus*): Diversity, incidence and persistence of infection at the individual level. *Parasites & Vectors* 16, 15. doi:10.1186/s13071-022-05623-x. 36641440 PMC9840293

[ref39] Svobodová M, Weidinger K, Peške L, Volf P, Votýpka J and Voříšek P (2015) Trypanosomes and haemosporidia in the buzzard (*Buteo buteo*) and sparrowhawk (*Accipiter nisus*): Factors affecting the prevalence of parasites. *Parasitology Research* 114, 551–560. 25403377 10.1007/s00436-014-4217-x

[ref40] Synek P, Popelková A, Koubínová D, Šťastný K, Langrová I, Votýpka J and Munclinger P (2016) Haemosporidian infections in the Tengmalm’s Owl (*Aegolius funereus*) and potential insect vectors of their transmission. *Parasitology Research* 115, 291–298. 26365667 10.1007/s00436-015-4745-z

[ref41] Valkiūnas G (2005) *Avian Malaria Parasites and Other Haemosporidia*. London: CRC Press.

[ref42] Valkiūnas G, Bairlein F, Iezhova TA and Dolnik OV (2004) Factors affecting the relapse of *Haemoproteus belopolskyi* infections and the parasitaemia of *Trypanosoma* spp. in a naturally infected European songbird, the blackcap, *Sylvia atricapilla*. *Parasitology Research* 93, 218–222. 15138804 10.1007/s00436-004-1071-2

[ref43] Venkatachalam AKSB, Čepička I, Hrazdilová K and Svobodová M (2023) Host specificity of passerine *Lankesterella* (Apicomplexa: Coccidia). *European Journal of Protistology* 90, 126007. doi:10.1016/j.ejop.2023.126007. 37536235

[ref44] Ventim R, Tenreiro P, Grade N, Encarnaçao P, Araújo M, Mendes L and Ramos JA (2012) Characterization of haemosporidian infections in warblers and sparrows at south-Western European reed beds. *Journal of Ornithology* 153, 505–512.

[ref45] Votýpka J, Synek P and Svobodová M (2009) Endophagy of biting midges attacking cavity nesting birds. *Medical and Veterinary Entomology* 23, 277–280. 19531067 10.1111/j.1365-2915.2009.00800.x

[ref46] Wilkinson LC, Handel CM, Van Hemert C, Loiseau C and Sehgal RN (2016) Avian malaria in a boreal resident species: Long-term temporal variability, and increased prevalence in birds with avian keratin disorder. *International Journal for Parasitology* 46, 281–290. 26828894 10.1016/j.ijpara.2015.12.008

[ref47] Wood MJ, Cosgrove CL, Wilkin TA, Knowles SC, Day KP and Sheldon BC (2007) Within-population variation in prevalence and lineage distribution of avian malaria in blue tits, *Cyanistes caeruleus*. *Molecular Ecology* 16, 3263–3273. 17651202 10.1111/j.1365-294X.2007.03362.x

[ref48] Yang G, Peng Y, Wang H, Huang X and Dong L (2023) Nowhere to escape: The cross‐age avian haemosporidian exposure of migrants in northeast China. *Journal of Avian Biology* 2023, e03091. doi:10.1111/jav.03091.

[ref49] Yusupova DA, Schumm YR, Sokolov AA and Quillfeldt P (2023) Haemosporidian blood parasites of passerine birds in north-western Siberia. *Polar Biology* 46, 497–511.

[ref50] Zuk M and McKean KA (1996) Sex differences in parasite infections: Patterns and processes. *International Journal for Parasitology* 26, 1009–1024. 8982783

