# Supplementary material for: Factors influencing a common but neglected blood parasite prevalence in breeding populations of passerines
Source: Parasitology. 2025 Jan 27;152(2):172–8. doi: 10.1017/S0031182025000095 (PMC12089444; doi:10.1017/S0031182025000095)
Supplement: Saravana Bhavan Venkatachalam et al. supplementary material [file S0031182025000095sup001.docx]

**Supplementary**

**Fig. i.** *Lankesterella* prevalences of adult and juvenile warblers (*A. schoenobaenus* (ACRSCH), *A. palustris* (ACRPAL), and *A. scirpaceus* (ACRSCI)). Number of individuals are shown above the columns.

**Fig. ii.** *Lankesterella* prevalences in adult and juvenile tits from Milovice forest (**M**) and Zeměchy (**Z**); (*C. caeruleus* (CYACAE), *P. major* (PARMAJ), and *P. palustris* (POEPAL)). Number of individuals are shown above the columns.

**Fig. iii.** *Lankesterella* prevalences in male and female adults of warblers (*A. schoenobaenus* (ACRSCH), *A. palustris* (ACRPAL), and *A. scirpaceus* (ACRSCI)). Number of individuals are shown above the columns.

**Fig. iv.** *Lankesterella* prevalences in male and female adult tits from Milovice forest (**M**) and Zeměchy (**Z**) (*C. caeruleus* (CYACAE), *P. major* (PARMAJ), and *P. palustris* (POEPAL)). Number of individuals are shown above the columns.
